# The LysR-Type Transcriptional Regulator YeeY Plays Important Roles in the Regulatory of Furazolidone Resistance in *Aeromonas hydrophila*

**DOI:** 10.3389/fmicb.2020.577376

**Published:** 2020-09-09

**Authors:** Yuying Fu, Lishan Zhang, Guibin Wang, Yuexu Lin, Srinivasan Ramanathan, Guidi Yang, Wenxiong Lin, Xiangmin Lin

**Affiliations:** ^1^Fujian Provincial Key Laboratory of Agroecological Processing and Safety Monitoring, School of Life Sciences, Fujian Agriculture and Forestry University, Fuzhou, China; ^2^Key Laboratory of Crop Ecology and Molecular Physiology (Fujian Agriculture and Forestry University), Fujian Province University, Fuzhou, China; ^3^State Key Laboratory of Proteomics, Beijing Proteome Research Center, National Center for Protein Sciences (Beijing), Beijing Institute of Lifeomics, Beijing, China; ^4^Key Laboratory of Marine Biotechnology of Fujian Province, Institute of Oceanology, Fujian Agriculture and Forestry University, Fuzhou, China

**Keywords:** *Aeromonas hydrophila*, DIA based proteomics, YeeY, furazolidone, antimicrobial resistance

## Abstract

*Aeromonas hydrophila* is an aquatic pathogen of freshwater fish. The emergence of widespread antimicrobial-resistance strains of this pathogen has caused increasing rates of fish infections. Our previous research reported that *A. hydrophila yeeY*, a LysR-type transcriptional regulator (LTTR), negatively regulated furazolidone (FZ) resistance. Although, it’s intrinsic regulatory mechanism is still unclear. In this study, a data-independent acquisition (DIA) quantitative proteomics method was used to compare the differentially expressed proteins (DEPs) between the Δ*yeeY* and wild-type strain under FZ treatment. When compared to the control, a total of 594 DEPs were identified in Δ*yeeY*. Among which, 293 and 301 proteins were substantially increased and decreased in abundance, respectively. Bioinformatics analysis showed that several biological pathways such as the secretion system and protein transport were mainly involved in FZ resistance. Subsequently, the antibiotics susceptibility assays of several gene deletion strains identified from the proteomics results showed that YeeY may regulate some important genes such as *cysD*, *AHA_2766*, *AHA_3195*, and *AHA_4275*, which affects the FZ resistance in *A. hydrophila*. Furthermore, 34 antimicrobial resistance genes (ARGs) from the bacterial drug resistance gene database (CARD) were found to be directly or indirectly regulated by YeeY. A subsequent assay of several ARGs mutants showed that Δ*AHA_3222* increased the susceptibility of *A. hydrophila* to FZ, while Δ*cysN* and Δ*AHA_3753* decreased the susceptibility rate. Finally, the chromatin immunoprecipitation (ChIP) PCR and an electrophoretic mobility shift assay (EMSA) have revealed that the genes such as *AHA_3222* and *AHA_4275* were directly and transcriptionally regulated by YeeY. Taken together, our findings demonstrated that YeeY may participate in antimicrobial resistance of *A. hydrophila* to FZ, which provides a new target for the development of novel antimicrobial agents in the future.

## Introduction

*Aeromonas hydrophila* is a well-known pathogen of freshwater fish, which is widely distributed in aquatic habitats including surface water, oceans, sewage and even chlorinated water (Liu et al., 2019). The number of reported cases caused by *A. hydrophila* has been grown worldwide. Therefore, this pathogen has received an increasing attention to combat their infections. Further, a several earlier studies have reported that *A. hydrophila* is a causative agent of hemorrhagic septicemia and motile aeromonad septicemia (MAS) in fish (Zahran et al., 2018; Abdelhamed et al., 2019). Outbreaks of *A. hydrophila* in humans have been recorded since 1992, which thought to be related to diarrhea, septicemia and soft-tissue wound infections in humans (de la Morena et al., 1993; Soltan Dallal et al., 2016). Further, a tons of antibiotics are used in aquaculture each year for controlling the infections in aquaculture worldwide, which causes a significant bacterial antibiotic resistance (Dahanayake et al., 2019). Therefore, the fish vaccines are used to the control the spread of diseases connected with *A. hydrophila*. However, only a few aquatic vaccines have obtained the national veterinary drug certifications in China, meaning that control measures are far from meeting the needs of the *status quo* (Wang Q. et al., 2020). Thus, it is necessary to understand the mechanism of antibiotic resistance in this pathogen to develop new and novel therapeutic agents.

It is generally agreed that the production of enzymes that inactivate antibiotics, changes in the target of antibiotics, increases in the permeability of cell membrane pore proteins and activation of efflux pumps are classic mechanisms of bacterial resistance (Das et al., 2020). Moreover, the previous studies have documented the outer membrane proteins (e.g., LamB and OmpA), fatty acid biosynthesis and the central metabolism of *A. hydrophila* also play an important roles in antibiotic resistance (Lin et al., 2014; Li et al., 2016; Yao et al., 2016). Nevertheless, specific mechanisms of antibiotic resistance are still largely elusive; although the proteins involved in antibiotic resistance yet need to be discovered.

LysR-type transcriptional regulators (LTTRs) are belonging to the transcription regulation families, which are widespread in prokaryotes. They are generally reported to be involved in bacterial antibiotic resistance. For example, the LTTR protein CidR was reported to positively regulate the *Staphylococcus aureus cidABC* operon, which enhances murein hydrolase activity and affects antibiotic tolerance against penicillin, vancomycin and rifampicin (Yang et al., 2005). A another LTTR protein OxyR regulates the cell division superfamily (RND) efflux pump gene *acrB* in *Klebsiella pneumoniae* and thereby confers resistance to chloramphenicol, erythromycin, nalidixic acid and trimethoprim (Rice et al., 2005). Since, LTTRs control diverse multi-functional genes and proteins as a well-known transcriptional regulator family, the antibiotic resistance regulatory functions of LTTRs are needed to be further investigated.

YeeY in *A. hydrophila* ATCC 7966 belongs to the LTTRs family that encodes a putative HTH-type transcriptional regulator. A multiple sequence alignment analysis showed that it’s DNA binding domain with an HTH motif at the N terminal and a regulatory domain at the C terminal is highly conserved with those of other LTTRs (Kim et al., 2018). Interestingly, the deletion of *yeeY* (*AHA_3980* as well) has displayed multidrug characteristics, which significantly increased resistance to chloramphenicol, chlortetracycline, ciprofloxacin, furazolidone and balofloxacin. So, it clearly suggests that the YeeY act as a negative transcriptional regulator during antibiotic stresses (Fu et al., 2019). However, the intrinsic antibiotic resistance mechanism regulated by *A. hydrophila* YeeY remains unclear.

In this study, to further understand the role of YeeY in antibiotic resistance, we compared the differentially expressed proteins (DEPs) between Δ*yeeY* and wild-type strains under the stress of FZ using data-independent acquisition (DIA) quantitative proteomics technology. The bioinformatics analysis showed that the deletion of *yeeY* affected several key biological processes and metabolic pathways. Several DEPs were subsequently validated by Western blotting and several related genes were deleted and their antimicrobial susceptibilities were evaluated. Furthermore, the outcome of chromatin immunoprecipitation (ChIP) PCR and electrophoretic mobility shift assay (EMSA) were suggested that some antimicrobial resistance related genes were directly and transcriptionally upregulated by YeeY. Overall, the obtained results of this study provide a novel understanding on the regulatory role of LTTRs YeeY in antibiotic resistance of *A. hydrophila* against FZ.

## Materials and Methods

### Bacterial Strain and Sample Preparation

*A. hydrophila* ATCC 7966 (WT), *yeeY* deleted strain (Δ*yeeY*), the rescue strain (Δ*yeeY* + *pACYC184-yeeY*), a negative control strain (Δ*yeeY* + *pACYC184*) and other gene deletion strains were constructed and stored in our laboratory as previously described (Pang et al., 2018). Bacterial strains were cultured overnight in Luria-Bertani (LB) medium at 30°C on a shaker at 200 rpm and then transferred to 100 mL LB medium at a ratio of 1:100 with a final concentration of 1.5 μg/mL of FZ treatment. The cells were collected by centrifugation at 8000 g for 15 min at 4°C when the OD of the bacteria culture reached 1.0 at 600 nm. After washed three times with pre-cooled PBS buffer, the cell pellets were resuspended in lysis buffer (6 M urea, 2 M thiourea, 100 mM Tris–HCl pH 8.5, protease inhibitor) and then ultrasonically disrupted by sonication on ice for 15 min at 30% power with intervals of 9 s. Then, the whole proteins were isolated by centrifuging at 12000 × *g* for 30 min at 4°C and the concentrations were determined by the Bradford method.

### Trypsin Digestion

A total of 50 μg protein of each sample was reduced with dithiothreitol (DTT), alkylated by iodoacetamide (IAA) and then digested with trypsin at a ratio of 1:50 for 16–18 h at 37°C. Subsequently, the digested peptides were desalted using a Sep-Pak Vac C18 Column (Waters Inc., Milford, MA) and dried using a CentriVap Concentrator (Labconco, Inc., Kansas City, MO) (Sun et al., 2019; Wisniewski, 2019; Yao et al., 2019).

### DIA Based LC-MS/MS Analysis

The digested peptides were resuspended in 25 μL of 0.1% formic acid (FA) containing iRT standard peptide (Biognosys) and then submitted to analysis on an Q Exactive HF mass spectrometer (Thermo Scientific, United States) with an EASY-nano-LC1200 chromatographic system (Thermo Scientific, United States) as previously described (Doerr, 2014). Briefly, peptides were loaded onto a C18 Trap column EASY-nano-LC at a rate of 4.5 μL/min with 12 μL phase A (2% acetonitrile and 0.1% FA) at a maximum pressure of 280 bars and then eluted using a C18 column with a runtime of 120 min at a rate of 600 nL/min using phase A (2% acetonitrile and 0.1% FA) and phase B (98% acetonitrile and 0.1% FA) with the following gradient elution program: 0–18 min, 6–12% B; 18–77 min, 12–20% B; 77–109 min, 20–32% B; 109–110 min, 32–90% B; 111–120 min, 90% B hold. The data were collected in the nano-spray ion source with an ion-spray voltage of 2.1 kV and an ion source temperature of 320°C. The scan range was 350 to 1400 m/z at a resolution of 60000 FWHM (at m/z 200) with automatic gain control (AGC) set to 3 E^6^ (maximum injection time of 20 ms). Further, the MS/MS was scanned at a resolution of 15000 FWHM (at m/z 200) with an isolation window of 1.6 Da with AGC set to 5 E^4^ (maximum injection time of 45 ms). DIA was scanned at a resolution of 30000 FWHM (at m/z 200) with a set of 45 variable overlapping windows covering the precursor mass range of 350–1400 m/z.

A confidential spectral library generated as previously study was used for spectral library (Wang G. B. et al., 2020). The DIA-generated data were imported into Spectronaut Pulsar X for protein qualitative and quantitative analyses using the following parameters: the iTR curve preceded local (non-linear) regression and protein identification was performed with Precursor Q value Cutoff 0.01 software. The identified proteins with at least two peptides matching and the peptide and protein FDR (false discovery rate) less than 0.01 were further analyzed.

### Bioinformatics Analysis

The Gene Ontology (GO) annotations and Kyoto Encyclopedia of Genes and Genomes (KEGG) pathways of DEPs were analyzed with DAVID online software^1^ (Li H. L. et al., 2020; Zhang et al., 2020). The GO and KEGG results were displayed in a diagram using GOCircle and GOChord plot functions of the GOplot R package. The predicted antibiotic resistance of DEPs was analyzed with the Comprehensive Antibiotic Research Database (CARD^2^) and visualized with loop heat map using the TBtool software (Chen et al., 2019, 2020).

### Western Blotting Validation

To validate the proteomics results, several specific antibodies were developed in house and Western blotting was performed as described previously (Li L. et al., 2020). Protein samples from each group were run on SDS-PAGE gels and transferred to PVDF membranes (Millipore, Billerica, MA, United States) for 15 min at 25 V using a Trans-Blot SD Cell (Bio-Rad, Hercules, CA, United States). After that, the PVDF membranes were washed three times with PBST and blocked in 5% skim milk with PBS buffer containing 0.05% Tween 20 (PBST) for 1 h and then probed with primary antibody before being incubated for 1 h at room temperature. Then, the membranes were washed with PBST five times and incubated in HRP goat anti-mouse or anti-rabbit IgG for 1 h at room temperature. Finally, the PVDF membranes were washed five times with PBST, exposed using the ECL system and visualized with a ChemiDoc^TM^ MP imager (Bio-Rad, Hercules, CA, United States). The band signals were quantified with Image J software.

### Antibiotics Susceptibility Assay

The antibiotics susceptibility assay was performed as described previously (Yao et al., 2018; Zhang et al., 2019). Briefly, the mutant and WT strains were incubated at 30°C for overnight and then diluted to ratio of 1:100 with fresh LB medium. After being treated with a series of concentrations of 0.25, 0.5, 1.0, and 2.0 μg/mL of FZ, the samples were divided into a HONEYCOMB^®^ Sterile 100 well plate and the OD_600_ value was determined at 16 h using a Bioscreen C instrument (Oy Growth Curves AB Ltd., Helsinki, Finland).

### ChIP-PCR

To further investigate the regulations between transcriptional regulator YeeY and candidate genes, ChIP-PCR was performed as previously described with slight modifications (Shen et al., 2020). Briefly, Δ*yeeY* containing *pACYC184-His-yeeY* or *pACYC184-His* were incubated at 30°C for overnight and then collected by centrifugation at 6000 g for 10 min at 4°C. After being washed three times with pre-cooled PBS buffer, the pellets were resuspended in 40 μL of 1% formaldehyde containing PBS buffer and placed on ice for 5 min; then, 3 M glycine solution was added for 15 min to terminate the crosslinking reaction. After crosslinking, the chromatin was collected and resuspended in 40 μL PBS buffer. After that, the chromatin was fragmented by sonication on ice for 20 min at 30% power with intervals of 9 s on ice to obtain an average length of 300 to 500 bps. Then, the nickel beaver beads were incubated with the crosslinking products for 2 h at 4°C and then the immunoprotein was eluted using 400 μL of 500 mM imidazole. After immunoprecipitation, 10% SDS and 2 μL of 100 mg/mL proteinase K was added to the precipitate and the samples were stored at 37°C. Next, the crosslinking was reversed and the DNA was extracted using phenol, chloroform and isoamyl alcohol at a ratio of 25:24:1 and then dissolved in 100 μL sterile water. Finally, the purified DNA was analyzed by ChIP-PCR using the primers listed in Table 1.

**TABLE 1:** Primer sequences used for ChIP-PCR and EMSA in this study.

**Primer**	**Oligonucleotide sequence (5′ → 3′)**	**Purpose**
*AHA_3222*-F	atgcggatcctgttggt	ChIP-PCR
*AHA_3222*-R	tcatgcccggtggtc	ChIP-PCR
P*_*AHA_*__3222_*-F	tgagcgcaacgcaataagcttTGTTATTCCCCTCAAAAGACGC	ChIP-PCR
P*_*AHA_*__3222_*-R	gagctgtacaagtaaggatccACCCAAGTCGGATCAGAGCG	ChIP-PCR
*AHA_4275*-F	ttgatatttgagacacttactatgattgc	ChIP-PCR
*AHA_4275*-R	ttaccagcggtagttgatgc	ChIP-PCR
P*_*AHA_*__4275_*-F	tgagcgcaacgcaataagcttTGAGTTGTTTCTGATTTCTTATTATTGG	ChIP-PCR
P*_*AHA_*__4275_*-R	gagctgtacaagtaaggatccCAATGGGGATCCCCGATG	ChIP-PCR
*AHA_3222*-F2	gctatttaaactctgcccaccagcttgcatactggtgggcagcgtcttttgaggggaataaca-Cy5	EMSA
*AHA_3222*-R2	tgttattcccctcaaaagacgctgcccaccagtatgcaagctggtgggcagagtttaaatagc	EMSA
*AHA_3222*-F3	gctatttaaactctgcccaccagcttgcatactggtgggcagcgtcttttgaggggaataaca	EMSA
*AHA_4275*-F2	tagcatgtcgctgctttattcgcctgaggaaccgggctaccggcatgaccgggcaccaataataa gaa-Cy5	EMSA
*AHA_4275*-R2	ttcttattattggtgcccggtcatgccggtagcccggttcctcaggcgaataaagcagcgacatgcta	EMSA
*AHA_4275*-F3	tagcatgtcgctgctttattcgcctgaggaaccgggctaccggcatgaccgggcaccaataataagaa	EMSA

### EMSA

To investigate the binding of YeeY to the target genes’ promoters, the EMSA was performed as described previously (Heravi and Altenbuchner, 2014; Cheng et al., 2019). First, the recombinant proteins of pET-32a-YeeY were obtained via a prokaryotic expression system. Briefly, the *pET-32a* plasmid containing *yeeY* was transformed into *E. coli* BL21 (DE3). The recombinant proteins were induced with IPTG (isopropyl β-D-1-thiogalactopyranoside, 0.05 mM) at 30°C for 6 h and then purified using Ni-TNA column as described in previous study (Li et al., 2017). Second, two DNA probes were synthesized by PCR using two pairs of single-strand primers (Table 1) in which the upstream primer was labeled by Cy5-labeled oligonucleotides at the 5′ end, whereas two cold probes as a competitor were synthesized with the primers without the Cy5-labeled at the upstream primer 5′ end (Table 1). To ensure the specificity of the probes, we randomly selected pET-32a-Hcp, which was kept in our laboratory as a negative control and then the recombinant proteins pET-32a-YeeY and pET-32a-Hcp were incubated with probe and binding buffer (1 M pH 7.5 Tris–HCl, 5 M NaCl, 1 M KCl, 1 M MgCl_2_, 0.5 M pH 8.0 EDTA, 10 mg/mL BSA), respectively for 30 min at 4°C in the dark. Finally, the mixture was separated by PAGE gels and then scanned using Odyssey@ CLX (LI-COR, United States).

## Results

### The Deletion of *A. hydrophila yeeY* Increases the Antibiotics Resistance to FZ

**FIGURE 1:**
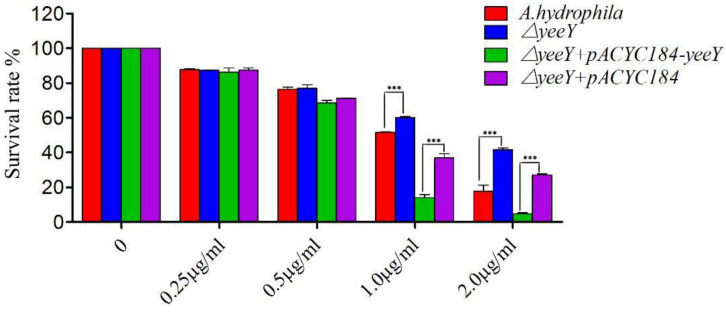
The antibiotics susceptibility of Δ*yeeY* derivatives to FZ. The survival rates of Δ*yeeY*, wild-type, Δ*yeeY* carrying *pACYC184*-*yeeY* (rescued strain) and Δ*yeeY* carrying an empty vector when exposed to various FZ concentrations.

The antimicrobial susceptibility rate of deletion and rescued strains were assessed when challenged with a series of FZ concentrations by a percentage of survival (Figure 1). At lower FZ concentrations (0.25 and 0.5 μg/mL), no significant differences were observed in the susceptibility rate of Δ*yeeY* or Δ*yeeY* + *pACYC184* when compared to the wild-type and Δ*yeeY* + *pACYC184-yeeY* strains, respectively. However, at the higher doses of FZ (1 to 2 μg/mL), the Δ*yeeY* strain showed significantly increased susceptibility rate, whereas the rescued strain showed decreased susceptibility rate when compared to the controls.

### Quantitative Proteomics of DEPs Between WT and △*yeeY* in Response to FZ

**FIGURE 2:**
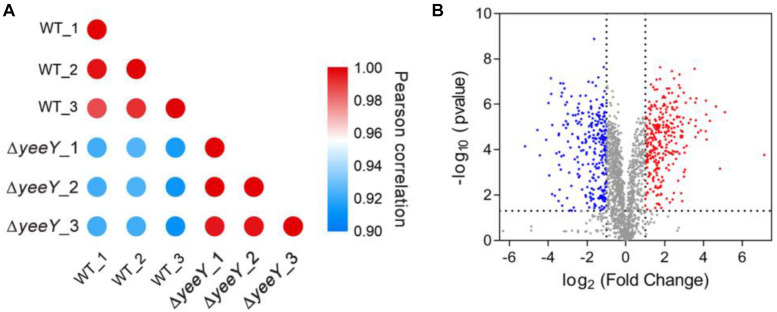
DIA based quantitative proteomics data analysis. **(A)** Correlation coefficients were used to analyze the associations of protein strength in three biological replications; **(B)** Volcano map comparing the abundance ratios of proteins with significant differences in expression (Fold change > 2; *p* < 0.05). The blue dots in the figure represent differentially down-regulated proteins; the red dots represent differentially increasing abundance proteins and the gray color represents non-differentially expressed proteins.

To further understand the effects of *yeeY* deletion on protein expression in *A. hydrophila*, whole protein samples between WT and Δ*yeeY* under 1.5 μg/mL of FZ antibiotic stress were extracted and then digested to peptides by trypsin to quantify in the protein levels by using DIA-based proteomics method. Each sample was independently repeated three times as biological replicates. Consequently, a total of 2066 proteins were identified with a considerable conservative threshold (protein and peptide false discovery rate <1%; listed in Supplementary Table S1) by LC-MS/MS. The protein MS intensities of WT and Δ*yeeY* were highly correlated among biological replicates (Pearson correction factor > 0.9) suggesting that the quantification analysis had high reliability (Figure 2A). Besides, as shown in the volcano plot, 593 DEPs were identified, including 293 increased and 300 decreased in abundance with fold change (FC) > 2 and *p*-value < 0.05 (Figure 2B).

### Go Enrichment of DEPs Under FZ Stress

**FIGURE 3:**
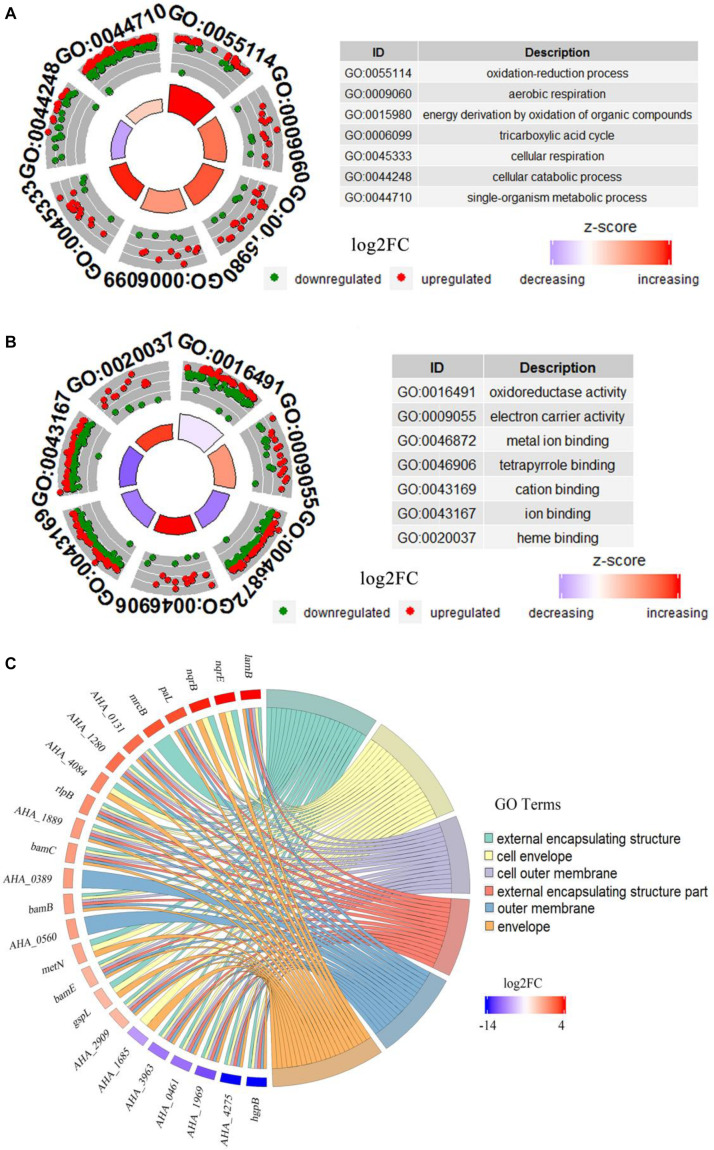
Bioinformatics analysis of DEPs between WT and Δ*yeeY* under FZ stress. **(A)** The cluster analysis of GO annotation in biological processes; **(B)** The cluster analysis of GO annotation in molecular functions; **(C)** The chord chart analysis of GO annotation in cellular components.

GO analysis of DEPs between WT and Δ*yeeY* strains under FZ stress was performed with DAVID and visualized using the GOplot package in the R software. In the biological process (BP) classification clusters, the significantly enriched GO terms were oxidation-reduction process (5.6%, 25 increased and 8 decreased in abundance), aerobic respiration (2.5%, 11 increased and 4 decreased in abundance), energy derivation by oxidation of organic compounds (3.7%, 17 increased and 5 decreased in abundance), tricarboxylic acid cycle (2.2%, 9 increased and 4 decreased in abundance), cellular respiration (3.2%, 15 increased and 4 decreased in abundance) and cellular catabolic process (5.4%, 9 increased and 23 decreased in abundance) (Figure 3A). In the molecular functioning (MF) classification clusters, the significantly enriched GO terms were oxidoreductase activity, electron carrier activity, metal ion binding, tetrapyrrole binding and cation binding. Of these clusters, many proteins were increased in abundance in tetrapyrrole binding, electron carrier activity and heme-binding (Figure 3B). In the cell component (CC) classification clusters, the most significantly enriched GO terms were external encapsulating structure, cell envelope, cell outer membrane, external encapsulating structure part and outer membrane and envelope. Further, the protein expression showed that *lamB* had the highest fold change, while the *hgpB* exhibited the lowest expression in the CC enrichment (Figure 3C).

### KEGG Enrichment of DEPs in FZ Stress

**FIGURE 4:**
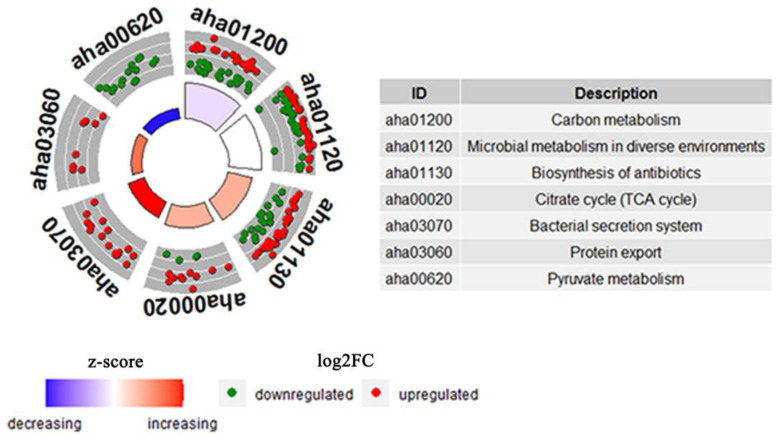
The cluster enrichment analysis of KEGG metabolic pathway of DEPs between WT and Δ*yeeY* under FZ stress. The three circles from outside to inside are indicated GO terms; a scatter plot of the expression levels (log_2_FC) for the genes in each, where red dots represent increasing abundance while green dots represent down-regulated; and a bar plot where the bar height indicates the significance of the KEGG pathway (log_10_p_value)_ and the color indicates the z-score gradient.

KEGG pathways enrichment analysis was further performed for the DEPs between the WT and △*yeeY* under FZ stress. The results showed that the most representative pathways were carbon metabolism (19 proteins increased and 23 proteins decreased in abundance), microbial metabolism in diverse environments (29 proteins increased and 29 proteins decreased in abundance), biosynthesis of antibiotics (33 proteins increased and 21 proteins decreased in abundance), TCA cycle (11 proteins increased and 3 proteins decreased in abundance), bacterial secretion system, protein export and pyruvate metabolism (Figure 4). Meanwhile, many pyruvate metabolism-related proteins were decreased in abundance, whereas bacterial secretion system and protein export-related proteins were found to be largely enriched with increased abundance. In general, most of these proteins are involved in key metabolic functions and protein export and these processes may contribute to FZ resistance.

### Western Blotting Validation of Proteomics Results

**FIGURE 5:**
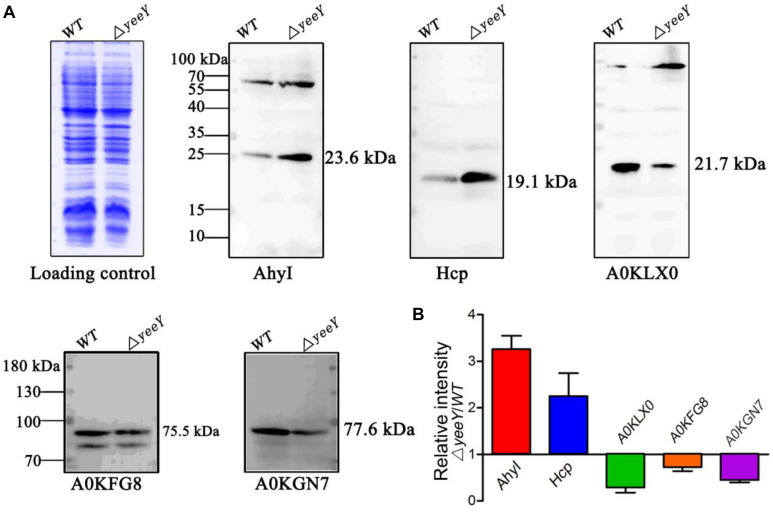
Western blotting analysis of the selected DEPs between WT and Δ*yeeY* under FZ stress. **(A)** The CBB-stained SDS-PAGE map of *A. hydrophila* WT and Δ*yeeY* under 1.0 μg/ml FZ treatment on the left acted as a loading control; **(B)** Gray level analysis of western blotting band signals by image J software.

To validate the proteomics results, two increased abundance proteins (AhyI and Hcp) and three decreased abundance proteins (A0KGN7, A0KLX0 and A0KFG8) were selected and analyzed by Western blotting using the primary antibodies that were previously developed by our group. The Western blotting results showed that Acyl-homoserine lactone synthase (AhyI) and hemolysin co-regulated protein (Hcp) were increased in abundance in Δ*yeeY*, while oligopeptide ABC transporter (A0KFG8), phosphate acetyltransferase (A0KGN7) and cytochrome c553 (A0KLX0) were down-regulated in Δ*yeeY*, compared to the WT strain under FZ stress (Figure 5A). All experiments were repeated at least three times, then the target bands intensity were quantified using Image J software and visualized with histogram (Figure 5B). Our data showed that the Western blotting results were relatively consistent with the mass spectrometry data, which indicates that our proteomics results were reliable.

### Survival Capability Assay of Related Mutants in Response to FZ Stress

**FIGURE 6:**
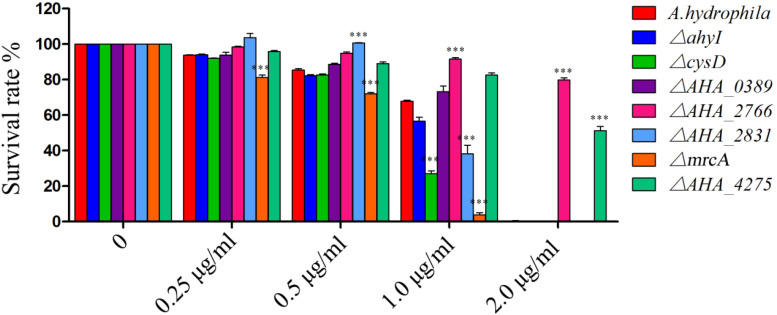
Survival capability assay of selected gene deleted strains under different doses of furazolidone treatment. ^∗∗∗^means *p* < 0.001.

To further understand the roles of YeeY on the altered proteins under FZ resistance, the antimicrobial susceptibilities of seven deletion mutants (Δ*ahyI*, Δ*cysD*, Δ*mrcA*, Δ*AHA_0389*, Δ*AHA_2766*, Δ*AHA_2831* and Δ*AHA_4275*), which were stored in our laboratory, were evaluated based on survival rate in a series of FZ concentrations treatment. Among these, *cysD*, *mrcA* and *AHA_4275* genes were involved in metabolic pathways and microbial metabolism in diverse environments. Further, the *cysD* gene was also involved in the biosynthesis of antibiotics and *AHA_2766* was involved in the part of metabolic pathways. The results showed that Δ*AHA_2766* and Δ*AHA_4275* had significantly increased survival rate, while Δ*cysD* and Δ*mrcA* had decreased survival rate when compared to the survival rate of the wild-type strain. Although *ahyI* and *AHA_0389* were showed increasing abundance in the proteomics results, whereas these two deleted mutants were showed no significant differences in survival rates, compared to the WT strain. Interestingly, Δ*AHA_2831* was showed increased survival rate in lower concentrations of antibiotics (0.25 to 0.5 μg/mL), while it was showed sharply decreased survival rate in 1.0 μg/mL of FZ. In general, our results indicated that *AHA_2766*, *AHA_4275*, *mrcA* and *cysD* may be regulated by YeeY and may be involved in FZ resistance (Figure 6).

### The Roles of CARD Drug-Resistant Genes on FZ Resistance

**FIGURE 7:**
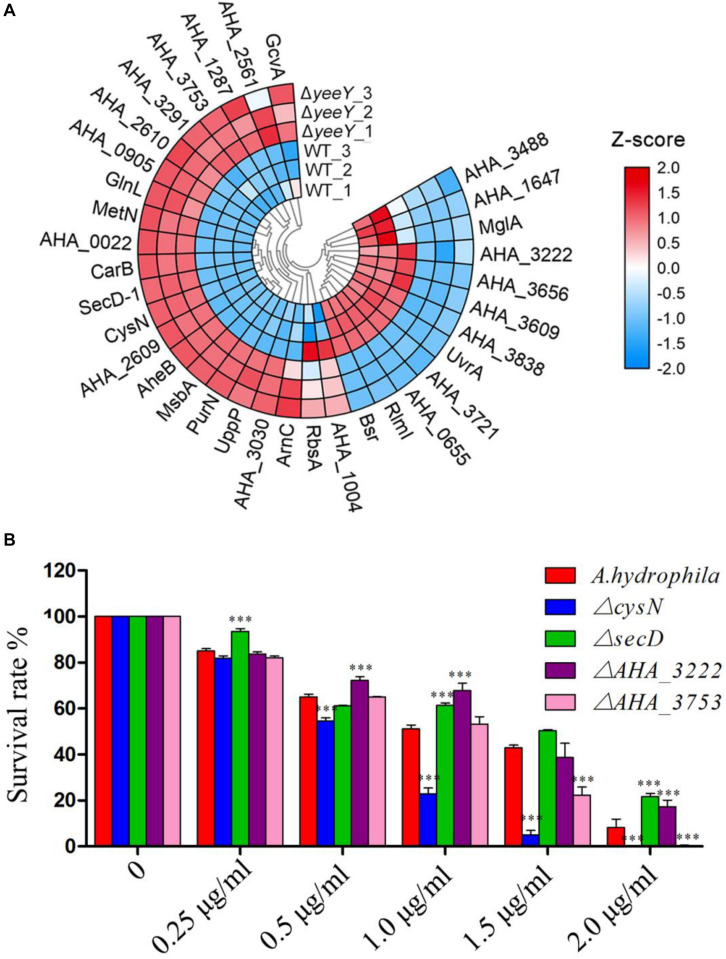
CARD ARGs analysis of DEPs between WT and Δ*yeeY* under FZ stress. **(A)** Loop heat map analysis of ARGs in Δ*yeeY* differentially expressed genes. The colors of squares indicate the z-scores, red represents increased and blue represents decreased. The outer cycle of the heat map represents the gene names of DEPs in Δ*yeeY*; **(B)** survival capability assay of four ARG deleted strains under different doses of furazolidone treatment.

To further understanding the effect of *A. hydrophila* YeeY on antimicrobial resistance genes (ARGs), all DEPs from proteomics analysis were submitted to ARGs detection by searching the CARD that provides well-known ARGs from diverse bacterial species. A total 30 resistance genes were found to be directly or indirectly regulated by YeeY under FZ stress, of which 22 and 12 proteins were increased and decreased in abundance, respectively (Figure 7A, Table 2). Subsequently, the antimicrobial susceptibilities of four resistance deletion mutants (Δ*cysN*, Δ*secD*, Δ*AHA_3222* and Δ*AHA_3753*), which were kept in our laboratory, were evaluated based on survival rate when treated with a series of FZ concentrations (Figure 7B). The results showed that the survival rates of Δ*AHA_3222* and Δ*secD* were increased while those of Δ*cysN* and Δ*AHA_3753* were significantly decreased under the stress of FZ. Therefore, our results indicated that the genes *cysN*, *AHA_3222* and *AHA_3753* were regulated by YeeY, which may participate in the resistance process of *A. hydrophila* to FZ.

**TABLE 2:** DEPs related to ARGs between Δ*yeeY* and WT *A. hydrophila* under FZ stress by DIA-LC-MS/MS.

**Accession**	**Gene**	**Description**	**Matched peptides**	***P*-value**	**log2(Δ*yeeY/WT*)**
A0KEF9	*tatA*	Sec-independent protein translocase protein TatA	3	5.43E-05	1.762
A0KEY7	*glnL*	Nitrogen regulation protein NR(II)	14	0.000149	2.754
A0KF41	*secY*	Protein translocase subunit SecY	7	1.43E-07	2.724
A0KG12	*AHA_0655*	Arginine ABC transporter, ATP-binding protein	9	4.24E-07	–2.348
A0KGQ2	*AHA_0905*	Aerobic respiration control sensor protein	26	1.74E-05	3.720
A0KGR2	*metN*	Methionine import ATP-binding protein MetN	13	1.65E-05	1.348
A0KGY7	*arnC*	Undecaprenyl-phosphate 4-deoxy-4-formamido-L-arabinose transferase	11	0.008264	2.845
A0KHD7	*gcvA*	Glycine cleavage system transcriptional activator	11	0.03694	1.501
A0KHS7	*AHA_1287*	HlyD family secretion protein	15	0.000332	1.685
A0KJ22	*secD-1*	Protein translocase subunit SecD	28	3.11E-07	2.377
A0KJ23	*secF-1*	Protein-export membrane protein SecF	8	2.73E-05	2.495
A0KKF0	*rlmI*	Ribosomal RNA large subunit methyltransferase I	13	4.55E-06	–1.337
A0KLC0	*AHA_2561*	Transcriptional regulator	10	0.036697	1.818
A0KLG5	*bsr*	Broad specificity amino-acid racemase	18	4.43E-06	–2.302
A0KLG7	*AHA_2609*	Oligopeptide ABC transporter, ATP-binding protein OppF	19	1.4E-05	1.330
A0KLG8	*AHA_2610*	Oligopeptide ABC transporter, ATP-binding protein OppD	14	0.000151	1.074
A0KLT0	*carB*	Carbamoyl-phosphate synthase large chain	52	9.59E-06	1.054
A0KLY2	*msbA*	Lipid A export ATP-binding/permease protein MsbA	22	9.88E-06	2.219
A0KM25	*purN*	Phosphoribosylglycinamide formyltransferase	5	8.26E-06	1.522
A0KMB3	*aheB*	Efflux pump membrane transporter	30	8.17E-06	1.915
A0KMN1	*AHA_3030*	ABC transporter, CydDC cysteine exporter (CydDC-E) family, permease/ATP-binding protein CydC	16	0.000171	2.044
A0KN35	*mrcA*	Penicillin-binding protein 1A	31	2.64E-05	1.993
A0KN62	*AHA_3222*	DNA-binding response regulator	7	0.007093	–1.178
A0KND1	*AHA_3291*	DNA-binding response regulator	10	0.002697	1.812
A0KNE6	*secG*	Protein-export membrane protein SecG	3	0.000126	2.184
A0KNW1	*AHA_3488*	ABC transporter, ATP-binding protein	5	0.036196	–1.357
A0KP35	*cysN*	Sulfate adenylyltransferase subunit 1	25	1.62E-06	2.398
A0KP36	*cysD*	Sulfate adenylyltransferase subunit 2	11	1.7E-05	2.395
A0KP78	*AHA_3609*	Transcriptional regulator, MarR family	6	0.000606	–1.397
A0KPB1	*AHA_3656*	Chloramphenicol acetyltransferase	3	3.86E-05	–1.009
A0KPG8	*AHA_3721*	Transcriptional regulator, MarR family	8	1.96E-05	–1.082
A0KPK0	*AHA_3753*	LysR-family transcriptional regulator	7	0.000149	1.924
A0KPT0	*AHA_3838*	Chemotaxis protein CheV	14	1.94E-05	–1.429
A0KQ38	*uvrA*	UvrABC system protein A	48	7.36E-05	–1.032
A0KQI1	*uppP*	Undecaprenyl-diphosphatase	2	8.05E-05	2.085
A0KQZ1	*AHA_4275*	Ferrichrome receptor	15	4.47E-07	–3.322
A0KQZ7	*yidC*	Membrane protein insertase YidC	26	3.61E-06	2.757
A0KR20	*AHA_0022*	RND transporter, hydrophobe/amphiphile efflux-1 (HAE1) family, MFP subunit	15	1.06E-05	1.720

### *AHA_3222* and *AHA_4275* Were Directly Regulated by YeeY in FZ Resistance

**FIGURE 8:**
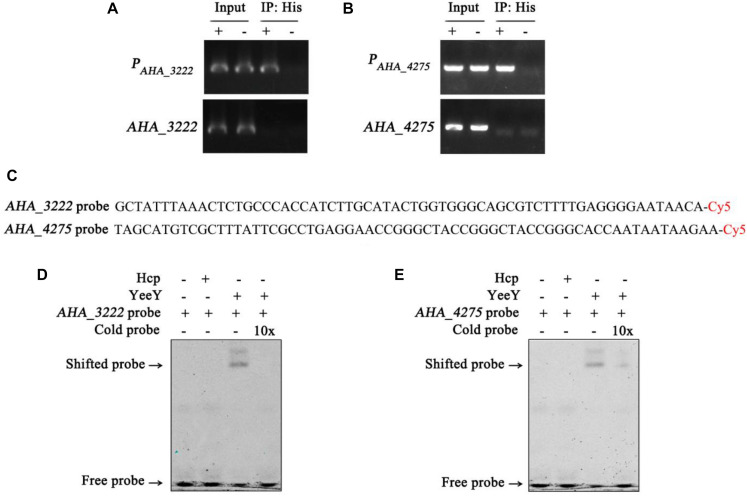
The binding of YeeY with the promoters of *AHA_3222* and *AHA_4275*. **(A,B)** ChIP-PCR analysis of the binding of YeeY and the promoter fragments of *AHA_3222* and *AHA_4275*. The promoter fragments of both genes are marked as P*_*AHA_*__3222_* and P*_*AHA_*__4275_*, respectively; **(C)** the nucleotide sequences of *AHA_3222* and *AHA_4275* DNA probes; **(D,E)** EMSA analysis of the binding of YeeY and the promoter fragments of *AHA_3222* and *AHA_4275*, respectively; The Cold probes competition were performed with 10 × unlabeled probes.

The data of this study indicates that YeeY may affect the FZ resistance of *A. hydrophila* via regulating the expression of some proteins, such as *cysN*, *mrcA*, *AHA_3222* and *AHA_4275*. Therefore, we have validated this possibility using ChIP-PCR technique. The *A. hydrophila* chromatin was isolated and immunoprecipitation with anti-His antibody and the acquired recovered DNA was used as a template for PCR with the target gene and its predicted promoter region primer pairs. The results showed that only the predicted promoter regions of *AHA_3222* and *AHA_4275* produced product in the Δ*yeeY*: *pACYC184-His-yeeY* IP sample, whereas *AHA_3222* and *AHA_4275* could not be amplified (Figures 8A,B). So, it is suggesting that *AHA_3222* and *AHA_4275* may be directly targeted by YeeY. To further confirm these results, EMSA was performed to investigate the binding of YeeY to *AHA_3222* and *AHA_4275* promoters *in vitro*. The results showed that promoter fragments of both genes were bound by YeeY (Figures 8C–E). Therefore, the outcome of EMSA clearly suggested that *AHA_3222* and *AHA_4275* can be directly regulated by YeeY.

## Discussion

*A. hydrophila* is an important aquatic bacterial pathogen in freshwater aquaculture and it has been reported that the threat of infection caused by this pathogen in fish and even in humans is on the rise worldwide (Awan et al., 2018). Although, the antibiotic treatment is an effective tactic to control the bacterial diseases in aquaculture, side however the effects such as antibiotic residue and antibiotic resistance cannot be ignored. Further, more than two million people who are infected with antibiotics resistant bacterial pathogens each year in the United States on average 23000 people die, meaning that antibiotic resistance has become one of the greatest threats faced by modern medicine (Laxminarayan et al., 2013). Therefore, elucidating the antibiotic resistance mechanism of *A. hydrophila* is of great significance for the development of new antibiotic therapeutic strategies in the future. YeeY, an LTTR, was reported to be associated with the rapid emergence of persistence in *E. coli* (Girgis et al., 2012). However, the biological function of this protein is still largely unknown, especially concerning antibiotic resistance. Although furazolidone has been prohibited in many parts of the world due to its toxic and carcinogenic side effects, we found that the minimal inhibitory concentration (MIC) of FZ was decreased when the YeeY was overexpressed and the absence of *yeeY* led to an increase the FZ resistance in our previous work (Fu et al., 2019). To further understand the biological behavior of YeeY on antibiotic resistance, differentially expressed proteins between Δ*yeeY* and WT strains were compared under FZ stress by DIA-quantitative proteomics in this study. A total of 594 DEPs were identified in Δ*yeeY*, with 293 proteins that were increased in abundance and 301 were decreased in abundance.

Of these DEPs, 15 outer membrane proteins were affected by the loss of *yeeY* under the FZ stress when compared to the wild-type strain. Among them, the proteins such as LamB, OprM, and OmpA were increased in abundance, whereas A0KQZ1 (*AHA_4275*) and A0KN35 (*AHA_2766*) were decreased in abundance. The OmpA has been reported to play an important role in the transport of antibiotics. For example, the MICs of chloramphenicol, aztreonam and nalidixic acid were decreased when the *ompA* was disrupted in *A. baumannii* (Smani et al., 2014). Further, in our previous study, we have observed that the MBCs (minimum bactericidal concentrations) of ceftriaxone sodium, apramycin, neomycin sulfate and gentamicin in Δ*AHA_2766* were increased more than 4-fold, whereas the MBCs of norfloxacin and chloramphenicol in Δ*AHA_4275* were decreased at least 2-fold (Li et al., 2019). Therefore, we have evaluated the antibiotic susceptibility of these both mutants to FZ antibiotic in this study. The survival test showed that the survival rates of Δ*AHA_2766* and Δ*AHA_4275* were significantly higher, compared to the wild strains under various doses of FZ. It suggests that *A. hydrophila* YeeY may regulate several antibiotic resistance-related outer membrane proteins during FZ stress.

MrcA (penicillin-binding protein 1a, PBP1a) and MrcB (penicillin-binding protein 1b, PBP1b) were both increased in abundance in Δ*yeeY* strain under FZ stress. Both proteins are essential for cell wall peptidoglycan biosynthesis and maintenance of cell growth. More both of these proteins are considered as antibiotic targets by beta-lactams (Kumar et al., 2012; King et al., 2017). Apart from their involvement in beta-lactams resistance, these both proteins were increased in abundance within the chlortetracycline (CTC) resistant strain in our previous study, whereas their actual functions in CTC resistance are still unknown (Li et al., 2018). In this study, the survival rate of Δ*mrcA* was decreased under high doses of FZ, which suggesting that the MrcA may be involved in FZ resistance as well. This finding indicates that YeeY may negatively regulate penicillin-binding protein against FZ stress.

The KEGG analysis showed that the bacterial secretion system was enriched in increased abundance proteins, including general secretion pathway (*gspC*, *gspG*), type II secretion system T2SS (*gspM*, *gspL*), type VI secretion system T6SS (*AHA_1826*, *AHA_1827*, *AHA_1840*, *AHA_1841*, and *AHA_1845*), Sec secretion system (*secD*, *yidC*, *secE*, *secF*, *secG*, *secY*, and *lepB*) and Tat-dependent system (*tatA*). Some studies have documented that the bacterial secretion system related proteins are involved in antibiotics resistance. For example, T6SS functioned not only as a virulence system, which also contributed to antimicrobial resistance in *A. baumannii* ATCC 19606, it was proved that upon the T6SS core component *vgrG* was deleted, the antimicrobial resistance to ampicillin, chloramphenicol and β-lactam antibiotics was altered (Wang et al., 2018). It indicated that T6SS may be closely related to furazolidone resistance in *A. hydrophila* since *AHA_1827* was highly homolog to *A. baumannii* ATCC 19606’s gene *vgrG* by comparative amino acid sequence analysis. Besides, beta-lactamase, a target of beta-lactam antibiotics, was transported through the Sec and Tat-dependent secretion systems (Pradel et al., 2009). Thus, protein transport may be an adaptive strategy in the FZ resistance in *A. hydrophila.*

The DEPs were also involved in complex cellular metabolic processes, especially the TCA cycle and sulfur metabolism. It was reported that the down-regulation of bacterial energy generation systems such as the TCA cycle may be a potential antibiotic resistance tactic in many bacterial species (Rosato et al., 2014; Peng et al., 2015; Su et al., 2018). However, many proteins associated with the TCA cycle, such as SucA, SucB, SucC, SucD, GltA, IcD, and SdhA were increased in abundance in this study, indicating that the role of the TCA cycle in FZ resistance in *A. hydrophila* may be alleviated by YeeY regulation. Sulfur, an essential element, exists in hundreds of metabolites with various oxidation states that are not only related to the virulence and antioxidant stress of *Mycobacterium tuberculosis* but also play important roles in rifampicin resistance. In the current study, four sulfur metabolism-related proteins (CysD, CysI, CysH, and CysN) were increased in abundance in Δ*yeeY* under FZ stress. The survival assay showed that the survival rate of Δ*cysD* and Δ*cysN* mutant strains were decreased when compared to the wild-type strain, which suggesting that sulfur metabolism is also a part of the mechanism of bacterial resistance by YeeY regulation. Taken together, these findings suggest that bacterial intracellular metabolism may be an important bacterial resistance strategy.

Also, we estimated the FZ susceptibilities of four well-known ARGs homologs (*AHA_3222*, *AHA_3753*, *cysN*, and *secD*) from the CARD by measuring the survival rates of their gene deletion strains under different concentrations of FZ. According to the annotation of CARD, *cysN* is related to tetracycline resistance; *AHA_3222* homolog is related to fluoroquinolone and acridine dye resistance*; AHA_3753* homolog is related to cephalosporin and *secD* is related to cephalosporin. There is considerable research documenting the antibiotic resistance properties of these ARGs or their homologs. For example, both SecD and SecF belong to the resistance-nodulation-cell division (RND) family of multidrug exporters and they are involved in the export of antimicrobial resistance proteins in *Staphylococcus aureus* (Quiblier et al., 2011). *AHA_3222* is a DNA-binding response regulator and its homologous protein ArlR (98% identity) in *S. aureus* was reported to positively regulate the expression of the efflux pump gene NorA and to be involved in bacterial multidrug resistance (Fournier et al., 2000); In addition to these, *Mycobacterium tuberculosis* sulfur metabolism genes including CysN were found to participate in certain antibiotics resistance as well (Hatzios and Bertozzi, 2011). In the current proteomics results, the protein level of *AHA_3222* (A0KN62) was decreased, while CysN and *AHA_3753* (A0KPK0) were increased in the Δ*yeeY* strain under FZ stress. Moreover, the survival of Δ*AHA_3222* was increased, while those of Δ*cysN* and Δ*AHA_3753* were decreased. It indicates that YeeY may positively regulate *AHA_3222* and negatively regulate *cysN* and *AHA_3753* by direct or indirect pathways contributing to survival under FZ stress. In addition, we found SecD was increased in the proteomics analysis, whereas the deletion of *secD* showed increased survival rate under FZ stress. This apparent inconsistency could be the result of a tradeoff between adaption and survival. As an important component of the Sec system for protein secretion, the deletion of *secD* could sharply reduce the membrane permeability and thereby prevent antibiotics entry. However, the loss of some important proteins such as outer membrane proteins will be disadvantageous for survival in the long term. Thus, the regulation of *secD* should be complex or there may be other transcriptional regulators involved.

Additionally, ChIP-PCR and EMSA were performed to explore the relationships of the LTTR protein YeeY with those genes. Interestingly, we found that YeeY can directly regulate *AHA_3222*, which may regulate the expression of efflux pump-related genes involved in bacterial multidrug resistance. Further, it also can directly regulate the outer membrane related gene (*AHA_4275*) during FZ stress. In general, our results provide evidence that the YeeY protein can bind directly with certain ARGs’ promoters that contribute to FZ resistance in *A. hydrophila*.

## Conclusion

In this study, we found that *A. hydrophila* YeeY could directly and positively regulate the ARG such as *AHA_3222* and *AHA_4275* and could indirectly or directly regulate several drug resistance-related genes as well as genes involved in key energy biosynthetic pathways such as metabolism and the bacterial secretion system. Overall, the outcomes of this study gave a view to understand the complicated regulatory mechanisms of transcription factors on bacterial physiological functions. More, it provided a new target for the treatment of pathogenic bacteria and the development of new antimicrobial agents.

## Data Availability Statement

All datasets generated for this study are included in the article/Supplementary Material.

## Author Contributions

XL and WL conceived and supervised the project. YF constructed strains, performed the experimental work, and drafted the manuscript. LZ and GW contributed to quantitative proteomics and data analysis. YL, SR, and GY improved the manuscript. All authors reviewed and approved the final manuscript.

## Conflict of Interest

The authors declare that the research was conducted in the absence of any commercial or financial relationships that could be construed as a potential conflict of interest.

## Footnotes

1 https://david.ncifcrf.gov/

2 http://arpcard.mcmaster.ca

## Supplementary Material

TABLE S1 Identification and quantification results between WT and Δ*yeeY* using DIA method in 1.5 g/mL FZ stress.
